# Action Observation and Effector Independency

**DOI:** 10.3389/fnhum.2019.00416

**Published:** 2019-11-26

**Authors:** Sonia Betti, Marie Deceuninck, Luisa Sartori, Umberto Castiello

**Affiliations:** ^1^Department of General Psychology, University of Padova, Padova, Italy; ^2^Department of Experimental Psychology, Ghent University, Ghent, Belgium

**Keywords:** motor resonance, action execution-action observation, effector-independency, motor evoked potentials, transcranial magnetic stimulation, corticospinal excitability

## Abstract

The finding of reasonably consistent spatial and temporal productions of actions across different body parts has been used to argue in favor of the existence of a high-order representation of motor programs. In these terms, a generalized motor program consists of an abstract memory structure apt to specify a class of non-specific instructions used to guide a broad range of movements (e.g., “grasp,” “bite”). Although a number of studies, using a variety of tasks, have assessed the issue of effector independence in terms of action execution, little is known regarding the issue of effector independence within an action observation context. Here corticospinal excitability (CSE) of the right hand’s first dorsal interosseous (FDI) and abductor digiti minimi (ADM) muscles was assessed by means of single-pulse transcranial magnetic stimulation (spTMS) during observation of a grasping action performed by the hand, the foot, the mouth, the elbow, or the knee. The results indicate that observing a grasping action performed with different body parts activates the effector typically adopted to execute that action, i.e., the hand. We contend that, as far as grasping is concerned, motor activations by action observation are evident in the muscles typically used to perform the observed action, even when the action is executed with another effector. Nevertheless, some exceptions call for a deeper analysis of motor coding.

## Introduction

When considering the issue of effector independence, two studies are frequently cited for empirical support, Merton (1972) and Raibert (1997). Both of these studies provide samples of handwriting phrases, which were similarly executed with different muscle-joint effector systems. Many have interpreted these findings as evidence that the motor program representation is generalized (see Keele, 1981; Schmidt et al., 1988; Rosenbaum, 1990).

This observed affinity of style across different effectors suggests that the representation of handwriting may be independent of the muscular activations that guide the pen. It must be said, however, that some differences between the effectors in terms of the size of the end result were noticed. For example, writing with a pen taped to the foot results in a spatially bigger end product. Nevertheless, the individual characteristics of the writer’s motor plan (e.g., the penmanship) remain visible. It seems, then, that the writing patterns shared only the very highest and most abstract representation (Wright, 1990; Castiello and Stelmach, 1993).

Effector-independency in action execution has also been investigated for grasping actions (Castiello, 1997; Parma et al., 2011). In a seminal study by Castiello (1997), mouth and hand movements were compared in a task asking participants to grasp pieces of cheese of different sizes with either the hand or the mouth. The pattern of mouth aperture with respect to the size of the food was similar to that found for grasping the very same objects with the hand. Similarly, it has been shown that hand and lip apertures are similarly scaled according to the size of an object evoked by a flavor. Maximum hand and lip apertures were greater when the action toward a small target (e.g., strawberry) was preceded by a sip of a “large” (e.g., orange) than a “small” (e.g., almond) flavor solution. Conversely, maximum hand and lip apertures were smaller when the action toward a large visual target (e.g., apple) was preceded by the presentation of a “small” (e.g., strawberry) rather than a “large” (e.g., orange) flavor solution (Parma et al., 2011).

Altogether these findings support the evidence concerned with the presence of a unique motor plan underlying the act of grasping with-the-hand and with-the-mouth, suggesting that coordinated actions are subserved by the use of a common coordinating schema independently from the effectors involved. Similarly, it has been demonstrated that the same holds true for tool use. When performing an action (e.g., pounding a nail) with different effectors (i.e., hand, foot, elbow), spatiotemporal parameters characterizing the execution of the action are kept constant among effector (Osiurak et al., 2018). This suggests that general motor programs are applied when using tools with different body parts.

The effector-independent coding for movements is also evident at neural level (Castiello et al., 1999; Rijntjes et al., 1999; Jastorff et al., 2010; Heed et al., 2011, 2016; Lorey et al., 2014). To dissociate brain regions devoted to the implementation of movement parameters from those relevant to the chosen effector, Rijntjes et al. (1999) asked participants to write their signature with their dominant index finger and ipsilateral big toe, and determined those areas activated by both conditions using functional magnetic resonance imaging (fMRI). The results show that movement parameters for this highly trained movement are stored in secondary sensorimotor cortices of the extremity with which it is usually performed, i.e., the dominant hand, including dorsal and ventral lateral premotor cortices. These areas can be accessed by the foot and are therefore functionally independent from the primary representation of the effector.

In another study, participants were required to perform or imagine an action (grasping a sweet) with either the mouth or the hand while the brain was scanned (Castiello et al., 1999). When “polished” from the motor component (i.e., execution) the registered activity showed inferior parietal lobe (IPL) activations for both movements. The proposal here was that the IPL plays a pivotal role in the coding of general action patterns in humans and it is the repository for effector independent representations.

Support to this contention comes from a study in which neural activity during memory-guided eye, hand, and foot movements in human participants was measured (Heed et al., 2011). The results did not reveal any significant activation differences during the planning of hand and foot movements, except in the most anterior part of the posterior parietal cortex (PPC). This region showed a lateral-to-medial gradient for hand vs. foot movement planning. The limb-unspecific PPC regions were functionally connected with hand and foot motor regions. Thus planning-related activity across effectors considerably overlapped.

The issue of effector independency is not confined to action execution, but it extends to action observation. For instance, when volunteers were presented with video clips showing four different motor acts (dragging, dropping, grasping, and pushing) performed with different effectors (foot, hand, and mouth), the coding of observed motor acts differed between the premotor and the parietal cortex. In the premotor cortex, they clustered according to the effector used, whereas in the inferior parietal lobule (IPL), they clustered according to the type of the observed motor act, regardless of the effector. Of interest, these results also suggest that in the case of motor acts typically done with the hand, the representations of such acts are used as templates for motor acts executed with other effectors (Jastorff et al., 2010).

In line with this latter observation, Senna et al. (2014) showed that when participants viewed a typical hand action (grasping a pencil) performed by either a hand or a foot, hand motor evoked potentials (MEPs) increased not only during the observation of actions performed by the hand but also for grasping actions performed by the foot. This evidence confirms that motor activations by action observation occur in the muscles typically used to perform the observed action, even when the action is executed with another effector (see also Betti et al., 2015). This kind of “hand” template activation has also been shown in a study in which corticospinal excitability (CSE) of participants observing the opening and closing movements of the mouth and hand was measured (Finisguerra et al., 2015).

The current research was set up to provide further evidence regarding effector-independent processes during action observation with specific reference to the hand template. Is the hand a reference point for whatever effector taking possession of an object? The majority of studies have investigated motor acts performed with the hand or the mouth, two effectors intimately related at both neural (Matelli et al., 1985; Rizzolatti et al., 1988) and functional level (Gentilucci et al., 2001). Grasping a fork to nail a piece of food is usually followed by a mouth grasp for eating the food, consequently, the grasp command can be sent to different distal effectors to prepare a series of successive motor acts. Further the fact that effector independency occurs when hand and foot actions are observed might not be surprising given that from an evolutionary perspective certain types of grips involving the entire surface of either the hand or the foot are part of the behavioral repertoire of primates (Macfarlane and Graziano, 2009; Castiello and Dadda, 2018).

With this in mind, here we test how far effector independency—in terms of hand template—goes by asking participants to passively observe not only grasping actions performed with either the foot, the hand or the mouth, but also grasping actions performed with effectors which are “distant” as far as grasping is concerned, namely the elbow and the knee. Specifically we assessed MEPs of two hand muscles, the first dorsal interosseous (FDI) and Abductor Digiti Minimi (ADM), during the observation of the above mentioned grasping movements. If observing a grasping action performed by whatever effector calls for an involvement of hand grasp representation, then we should find general facilitation in hand muscles for all effectors. This would signify that the hand template comes into play whatever grasping effector is observed and would shed more definite light on the notion of effector independence for action observation. Conversely, if hand MEPs modulations are evident only for more grasp-related effectors, then we should find an increase in the MEP amplitudes only during a hand, mouth and foot grasp observation, but not for elbow and knee.

## Materials and Methods

### Participants

A total of 29 healthy subjects (15 females, mean age: 22.8, range: 19–31 years) participated in the study. All participants had normal or corrected-to-normal vision and were right-handed. Handedness was assessed with the use of an Italian adapted version of the Edinburgh handedness inventory, a 10-item questionnaire to determine expressed hand preference (Oldfield, 1971). Subjects were screened for neurological, psychiatric or medical problems. None had a contraindication to TMS (Rossi et al., 2009). Written informed consents were given prior to the experiment and all participants were naïve to the studies’ purpose. The experimental procedures were approved by the Ethical Committee of the University of Padova and conducted in accordance with the ethical standards of the Declaration of Helsinki. No discomfort was reported during TMS stimulation and MEP acquisition. A right-handed female (age 24) with a background in ballet has performed the different actions showed in the video-clips. She provided written informed consent for the recorded videos to be used in the experiment and to be published.

### Stimuli

**Figure 1:**
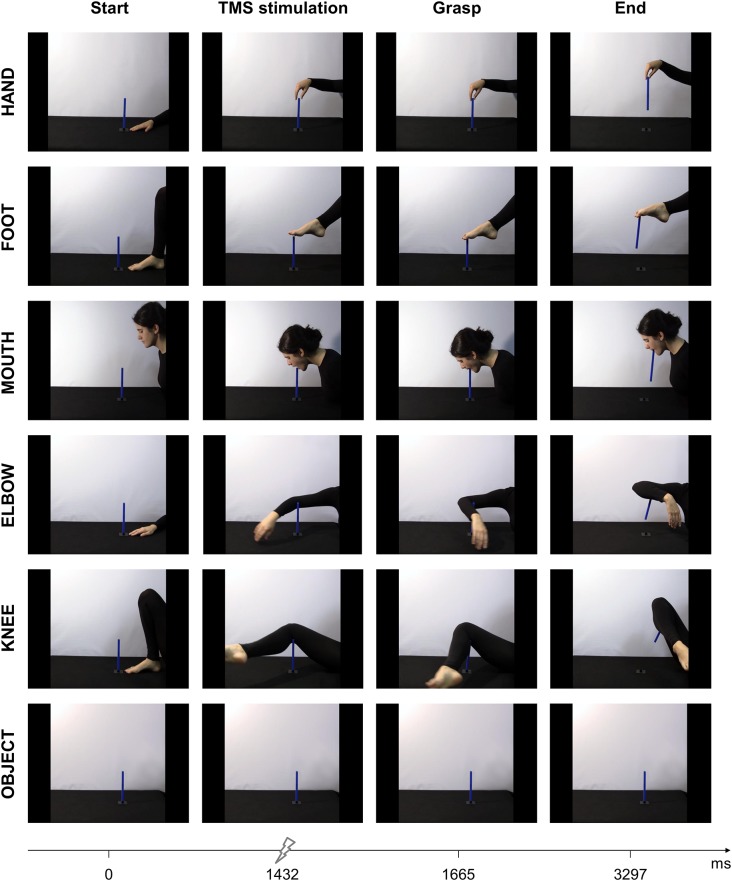
Sequence of events for the six experimental videos depicting a grasping action performed with different effectors: hand, foot, mouth, elbow, knee, and a control condition showing just the object. Each column represents an event, i.e., start of the action, TMS stimulation, contact with the object and end of action.

Six video-clips were used as experimental stimuli (Figure 1). The videos depicted a right-handed nonprofessional actress performing a grasping action with different effectors (hand, foot, mouth, elbow, and knee). The sixth video clip showed the object without any manipulation. The model was instructed to grasp the top of the object in a natural way and with the right-sided effectors. Furthermore, when grasping the object with the hand, the actress performed a pinch grasp. The object was a 3D printed rectangular parallelepiped (13 × 200 mm, 18 *g*) held uprights with the use of a small separate black platform (60 × 60 × 60 mm). The different video-clips were filmed from a lateral point of view with the use of a Canon Legria HFM36 (Tokyo, Japan) mounted on a tripod. They were later edited with Adobe Premiere Pro CS 5.5 software to minimize the visibility of other effectors unrelated to the performed action. All videos included the effector at rest in front of the object before the actual action, followed by a top grasp of the object and a straight upwards lift of the stick. The model was instructed to minimize any time variations between the start and the grasp. Each stimulus presentation lasted 3,297 ms and the animation effects were obtained by presenting each frame 33.3 ms in series. Notably, the first and last frames lasted 200 ms. The grasp occurred approximately 1,665 ms after video onset. The end of each action was decided to represent a similar object height. The dimension of each stimulus was 1,024 × 768 pixels displayed on a 24-inch monitor (resolution: 1,440 × 1,080 pixels, refresh rate 120 Hz, color depth: 32 bits). Each frame was presented in the center of the screen with a black background. The experimental task was designed and run with the use of E-prime software (Psychology Software Tools, version 2.0).

### Procedure

Subjects were instructed to sit down in a slightly raised armchair upon arrival. Their right arm was positioned on a cushion and their head was placed on a fixed headrest in the most comfortable way. They were instructed to keep their hand and head still and relaxed during TMS stimulation. The experiment was presented on a monitor at eye level, located 80 cm from the participant’s head. After scrubbing the skin on the points of interest on the right hand, the electromyography (EMG) was set up. TMS-induced MEPs were obtained from the FDI and ADM muscles of the participant’s right hand. After acquiring an accurate signal, the coil was fixed on its optimal position and a threshold value for primary motor cortex (M1) stimulation, i.e., resting motor threshold (rMT), was defined. Participants had the task to carefully observe the video clips presented on a monitor in front of them in random order. Between each video presentation, the participant was reminded to remain attentive to the video and as relaxed as possible. The experiment consisted of 120 single-pulse TMS and lasted approximately 25 min. Stimulation was given at 120% of the rMT. A total of 30 pre- and post-experiment stimulations (2 × 15) were used to acquire each participant’s baseline CSE. During baseline registration, each trial lasted 10 s and consisted of a black screen for 5 s followed by a white fixation cross (10 × 10 mm) for another 5 s. Stimulation was given during the latter. Furthermore, 90 TMS pulses (15 repetitions × 6 conditions) were given during each video clip presentation at 1,432 ms after video onset. This corresponds to seven frames before the actual contact point with the effector. As shown by Urgesi et al. (2010), higher motor facilitation can be found during the start and middle phases of a grasping action compared to the end phase. We, therefore, adopted a stimulation time that was anticipated with respect to the effector-object contact. An equal time frame was used for the object condition. To match the moment of stimulation for all grasping movements, both the start and end frames were prolonged (from 200 to 533 ms and from 200 to 800 ms, respectively). By adopting a variable duration for the first frame across conditions, effects due to anticipation of the stimulation timing are avoided. An interpulse interval of 10 s was applied to minimize possible carryover effects of the TMS pulse on the subsequent one.

### TMS and EMG

Single-pulse TMS was delivered with the use of a figure-eight coil (70 mm) connected to a Magstim BiStim^2^ Stimulator (Magstim Co., Whitland, UK). Stimulation was given to the hand region of the left M1. The coil was positioned tangentially to the scalp of the participant, with the handle pointing laterally and caudally, 45° from the midsagittal axis (Brasil-Neto et al., 1992; Mills et al., 1992). The optimal scalp position (OSP) was then determined by moving the coil in approximately 0.5 cm steps around the presumed area. Visual inspection of the MEPs of the right FDI and ADM, recorded through EMG, were used as feedback. More precisely, the location which elicited a maximal amplitude for both muscles was used as a hotspot. Once M1 OSP was obtained, the coil location was marked on a tight-fitting cap placed on the participant’s head. The optimal position of the coil was maintained still on the head with the use of a mechanical arm attached to a tripod. This position was checked continuously throughout the experiment. The rMT, i.e., the lowest stimulation intensity inducing peaks (≥50 μV peak-to-peak amplitude) in 50% of 10 trials in a relaxed muscle (Rossini et al., 1994), was found for each participant. rMT ranged from 32% to 50% (mean ± SD: 41.52 ± 4.39) of the maximum stimulator output for both muscles. Stimulation intensity was set at 120% of the individual’s rMT during the experimental session to ensure a stable and clear MEP signal.

MEPs of the right FDI and ADM muscle were recorded through pairs of Ag-AgCl surface electrodes (9 mm in diameter) placed in a belly-tendon montage. The right wrist was used for the ground electrode. Skin impedance was considered of good quality when it was below the 5Ω threshold level. This was assessed prior to the experimental session when the participant was at rest. The five electrodes were connected to an isolated portable ExG input box (Professional BrainAmp ExG MR, Munich, Germany). A twin-fiber optic cable transmitted the signals from the input box to the main EMG amplifier. The raw myographic signals were sampled at a 5 kHz rate, filtered and amplified before digitalization. Filtering occurred at a bandpass of 20 Hz-1 kHz and the data were stored on a computer for offline analysis. EMG activity was monitored during the stimulation to ensure relaxation in both muscles. To check for any EMG activity before TMS stimulation, pre-stimulus activity recordings of 100 ms were obtained. Any trials with an activation higher than 50 μV before TMS onset were discarded from the data to prevent any contamination of the MEP measurements. EMG data were collected up until 200 ms after TMS pulse.

### Post-experimental Questionnaire

A short questionnaire at the end of the experimental session was included to measure participant’s affinity with the actions. After presenting a picture depicting the moment of stimulation, three questions were asked. The participant had to respond to these questions on a five-point Likert scale. The order of the conditions was randomized between participants. First, the naturality of the action was inquired, followed by the probability of using this action and lastly, how many times they executed this action. The three questions were (as translated from Italian): (Q1) “How natural is the observed action to you?”; (Q2) “What is the probability that you would perform this action?”; and (Q3) “How many times do you usually perform this action?”

### Data Analysis

Peak-to-peak MEP amplitudes for the FDI and ADM muscles were recorded and analyzed offline using Brain Vision Analyzer software (Brain Products GmbH; Munich, Germany). All analyses were conducted on 25 of the 29 participants. Four participants were excluded from the analyses due to technical difficulties. MEP amplitudes were then averaged over each condition, for each participant. All deviations bigger or smaller than 2 standard deviations (*SD*) from the mean were removed from further analysis. A total of 10.23% of the trials were excluded as outliers; either due to pre-activation, no activation at all or because they exceeded 2 *SD*. For hand, foot, mouth, elbow and knee conditions, a mean (±SD) total of 12 ± 11%, 12 ± 11%, 8 ± 7%, 7 ± 5%, 11 ± 11% and 9 ± 8% of MEPs were excluded, respectively. The remaining MEP amplitudes for each subject were then normalized based on the participants’ baseline MEPs. A ratio was computed by dividing the mean MEP amplitude for each condition by the mean MEP amplitude obtained during pre- and post-baseline measurements (MEP_ratio_ = MEP_obtained_/MEP_baseline_).

First of all, a paired samples *t*-test between pre- and post-baseline MEPs was performed for each muscle individually. Second, a repeated-measures analysis of variance (ANOVA) was performed on the MEP ratios. Both muscle (FDI, ADM) and conditions (hand, foot, mouth, elbow, knee, object) were within-subjects factors. Effect size estimates were obtained using partial eta-squared ($\eta_{\text{p}}^{2}$). The sphericity of the data was verified prior to analysis. Mauchly’s test of sphericity indicated a violation on the assumption of sphericity ($\chi_{\text{condition}}^{2}$ = 31.07, *p* = 0.006; $\chi_{\text{condition*muscle}}^{2}$ = 27.47, *p* = 0.017). Greenhouse-Geisser estimates of sphericity (ε_condition_ = 0.67; ε_condition*muscle_ = 0.66) are used to correct for the degrees of freedom. A one-sample *t*-test against 1 on the normalized data was conducted to look for modulations compared to the baseline. We tested against 1 as this value represents equal activation between the baseline and condition as it is conducted on the normalized MEPs. To analyze the questionnaire responses, a one-way ANOVA on the mean score for the three questions (Q1, Q2, Q3) was conducted with the five grasping actions (hand, foot, mouth, elbow and knee) as within-subject factors (Norman, 2010; Sullivan and Artino, 2013). *Post hoc* pairwise comparisons were conducted using *t*-tests. A Bonferroni correction was used to counteract the problem of multiple comparisons, i.e., reducing the chance for a type-I error. Alpha levels for all statistical tests were set at 0.05.

## Results

No significant difference between the mean raw pre- and post-baseline MEP measurements in both muscles was found (ADM: *t*_(24)_ = 0.126, *p* = 0.90; FDI: *t*_(24)_ = 0.427, *p* = 0.809). Consequently, motor excitability before and after the experiment did not differ, which let us to conclude that any modulations in the MEPs are exclusively linked to our experimental conditions.

The ANOVA on the normalized MEP amplitudes showed a main effect of condition (*F*_(3.36,80.56)_ = 5.425, *p* = 0.001, $\eta_{\text{p}}^{2}$ = 0.184), indicating that observing different effectors elicits different MEP amplitudes in both hand muscles. Furthermore, a two-way interaction effect of muscle × condition (*F*_(3.3,79.23)_ = 4.708, *p* = 0.003, $\eta_{\text{p}}^{2}$ = 0.166) was found.

**Figure 2:**
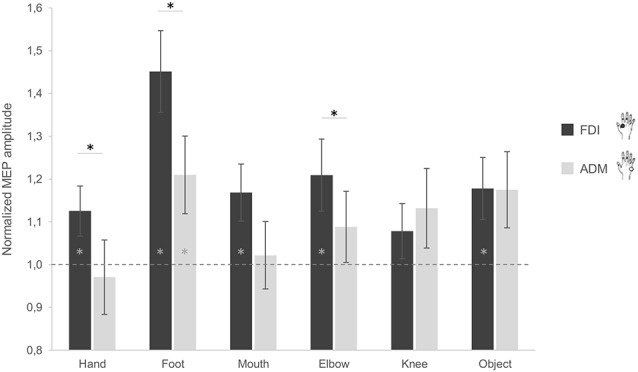
Effect of observing different effectors grasping a stick on corticospinal excitability (CSE) of the hand. MEP ratio modulations in the first dorsal interosseous (FDI; black) and abductor digiti minimi (ADM; gray) muscles. A value significantly different from 1 corresponds to facilitation (if positive) or inhibition (if negative) of the muscles as compared to baseline activation. Error bars represent standards errors and asterisks indicate statistically significant differences (*p* < 0.05).

When considering the difference between the FDI and ADM muscle activations, *post hoc* comparisons showed a significant difference during observation of grasping actions performed by the hand (*p* = 0.040), foot (*p* = 0.010) and elbow (*p* = 0.036). More precisely, the FDI muscle was significantly more activated compared to the ADM muscle during observation of grasping actions performed by these three effectors (Figure 2). For the mouth condition, the difference between FDI and ADM did not reach significance (*p* = 0.081). This higher FDI muscle elicitation during observation of a hand grasp was expected and suggest correct motor resonance.

Additionally, no significant difference between both muscles emerged while observing the object (*p* = 0.976). This is in line with the object affordance effect as the adopted object can be grasped with both a pinch grip (involving mainly the FDI) and a whole hand grasp (involving both muscles). Although not significant, an inverse activation of both muscles was instead found for the knee condition (*p* = 0.397). Plus, when considering the differences between conditions for the two muscles, *post hoc* analysis showed significant differences in the ADM between the hand and the foot (*p* = 0.011), and the hand and the object (*p* = 0.025), with lower ADM activation for the hand compared to the other conditions. For the FDI, a significant difference between the foot and the hand (*p* = 0.004), the foot and the mouth (*p* = 0.014), and the foot and the knee (*p* < 0.001) was found, with the foot condition having greater MEP amplitudes compared to the other effectors.

In terms of muscle facilitation with respect to the baseline condition, the FDI muscle showed an increased activation for all conditions, except the knee (*p*_s_ < 0.05; Figure 2). The ADM muscle was also significantly more activated during observation of the foot grasping an object compared to the baseline (*p* = 0.03; Figure 2). This is represented by having a significantly higher activation to one as analyses were conducted on ratios. These results show that the FDI muscle is generally activated during action observation, independently of the effector used in said observation.

Table 1 reports the mean scores of the post-experimental questionnaire, investigating how natural (Q1), probable (Q2) and frequent (Q3) is executing the observed grasping action with the different effectors. The ANOVA on the mean scores for the three items showed a main effect of condition (*F*_(4,96)_ = 68.741, *p* < 0.001, $\eta_{\text{p}}^{2}$ = 0.741). *Post hoc* comparisons showed higher scores were given to the hand action compared to all the other conditions (*p*_s_ < 0.05), and lower scores were given to the knee compared to all other effectors (*p*_s_ < 0.05).

**Table 1:** Mean (± SD) scores given to each item of the post-experimental questionnaire for each condition.

	Hand	Foot	Mouth	Elbow	Knee
Q1	4.48 ± 0.71	2.16 ± 1.11	2.28 ± 0.98	2.16 ± 1.18	1.48 ± 0.71
Q2	4.28 ± 0.79	1.96 ± 1.02	2.24 ± 1.05	1.92 ± 1.08	1.24 ± 0.52
Q3	3.80 ± 0.96	1.84 ± 1.07	2.24 ± 1.05	1.80 ± 1.12	1.08 ± 0.28

## Discussion

The present study was set up to address effector independency during action observation. By measuring CSE in the FDI and the ADM muscles during single-pulse transcranial magnetic stimulation (spTMS) on the primary motor cortex, we investigated if the hand, the effector typically used to perform a grasp, would present motor resonance only during observation of hand grasping or also while other body parts grasped an object, i.e., foot, mouth, elbow, and knee.

In general, the findings of this study point to an effector-independent activation of the motor system during action observation. CSE facilitation was evident in the effector usually adopted to perform a grasping action, namely the hand, even when the observed grasping was performed with a different body part. An explanation for this is that because the hand action templates were used to comprehend the goal of motor acts carried out using other effectors. It seems that one computational module is responsible for translating the sense of the same action performed with other effectors. This mechanism might imply the mapping of the observed action goal, which, in turn, might be functional to action understanding.

In this view, actions are abstractly encoded at a higher level in terms of its goal, regardless of the effector involved to achieve it. Indeed, when testing aplasic individuals who were born without arms and hands during observation of manipulative hand actions, overlapping activations emerged for foot and mouth action execution (Gazzola et al., 2007b). Even in the absence of a corresponding effector, these findings suggest that the mirror neuron system—matching action observation with action execution—is recruited for matching the observed action goal with the effector most frequently recruited to perform the action. Similarly, professional foot painters, who not only use their feet to compensate for missing hand function, but also achieved an extremely skilled and fine-grained control of their toe, showed a correspondence to canonical hand organization in their somatotopic toe map (Dempsey-Jones et al., 2019). This suggests that motor expertise and goal coding represent two critical aspects that can affect effector-independent motor representations.

Some authors have proposed that it is within the parietal cortex that motor acts are clustered according to the goal, irrespective of the effector used (Jastorff et al., 2010; Lorey et al., 2014). The recruitment of the muscles typically involved in the observed action found in our study, therefore, might reflect the parietal role in categorizing motor acts according to their functional meaning and in generalizing the observed actions across effectors. On the other hand, our typical sensory-motor experience, when considering grasping, implies that we typically take possession of objects with the hand.

Consistent with this interpretation are the findings that the human grasping circuit is strongly activated during the observation of grasping performed with artificial devices, even when the artificial device differs from a grasping hand in shape and kinematics (Gazzola et al., 2007a; Peeters et al., 2009). And they are also in line with previous evidence reporting generalized CSE for hand muscles during the observation of other effectors such as the hand and the foot grasping objects (Senna et al., 2014; Finisguerra et al., 2015).

A caveat of the present findings is that when observing foot grasping, the highest CSE modulations in both hand muscles was observed. A possible explanation for this result comes from action execution. As previously mentioned, writing with the foot determines an exaggeration of the writing size (e.g., Bernstein, 1967; Merton, 1972; Raibert, 1997). When we go more distal in our body schema, execution becomes more difficult and therefore less precise. As we are not skilled in writing with our foot, control over this distal body part is more difficult and requires more effort. Similarly, in action observation the mapping of the grasping action as performed by the foot might require more generalized activation to adapt the hand template to the foot grasping representation. Muscle activity may then reflect the neural parameters encoded in the motor program for actually executing or mentally performing the foot action.

Another aspect of the present results is that observing a knee grasp did not facilitate the targeted hand muscles. This lack of activation poses limits on the conclusion that it is only goal coding to determine effector independency for action observation. One of our hypotheses was that the “hand template” effect might be challenged by effectors which are “distant” as far as grasping is concerned. Indeed the knee was the most awkward to observe. This consideration is supported by data from our post-experimental questionnaire in which participants scored the knee action as the least natural, probable and frequent to execute. Therefore, not only the goal of an action but also how the observed action is feasible and it is part of our behavioral repertoire that allows accessing motor templates (e.g., Buccino et al., 2004; Gazzola et al., 2007b; Betti et al., 2015). In addition, action plausibility based on the available context may guide our processing of others’ actions. Along this line, Brass et al. (2007) investigated the role of mirror and non-mirror brain areas while observing goal-directed actions performed with an unusual effector (e.g., operating a light switch with the knee) in plausible (e.g., hand occupied by heavy folders) or implausible (e.g., hands-free) contexts. Results showed that presenting goal-directed knee actions did not activate mirror areas, rather the activation of the superior temporal sulcus was modulated by action plausibility, which the authors interpreted as a reflection of an inferential processing guiding action understanding. In our study, we did not look at contextual contingencies and constrains that could have justified the use of one effector with respect to another, still we found no motor activations for the knee grasping action. Overall, the results from Brass et al. (2007) support our findings, suggesting that the knee effector is hardly associated with goal-directed actions classically performed with the hands.

As a final aspect, one could argue that the heightened activation in the hand during the observation of other effectors performing a grasp is due to attention as the observed actions are rather unusual. However, the appropriate motor resonance response for the hand condition (muscle-specific activation for a pinch grasp with FDI > ADM, e.g., Cavallo et al., 2011) suggests that we are measuring CSE responses as a result of action processing. In addition, no difference between the FDI and ADM muscle was found for the object condition. As hypothesized, we did not expect to find a difference between these muscles, as the object is prone to both a pinch grasp and a whole hand grasp, the latter relying on both muscles. As previously found, the mere observation of an object should elicit activation in the muscles used to manipulate it (Cattaneo et al., 2005).

In conclusion, the present findings suggest that during the observation of grasping actions, the motor system is activated independently of the effector used by a model to perform the action. In particular, there was a tendency to match the observed action with its prototypical effector (i.e., the hand). This might simplify the understanding of action goals based on our experience. However, as witnessed by the lack of facilitation for the knee condition, this generalization process has some limits. If the effector used cannot be associated with a particular template, then there is no prototypical activation. This result bolds out the complexity of the involved mechanisms and calls for further experimentation to determine the boundaries of motor coding.

Overall, the existence of effector-independent action representations would allow us to flexibly map actions favoring the achievement of the underlying goal rather than the means to fulfill it. This would represent an advantage also in evolutionary terms: suppose that you are hungry and you find a nut, whether you crack it using your hand or your foot is irrelevant as long as you manage to eat it. During action observation, an effector-independent coding of the observed action would permit us to understand other’s goal-directed behavior, even in the presence of a non-canonical visual input. This applies, for example, for actions performed by people with motor impairments. In such circumstances the advantage is bidirectional: observers may easily understand goal-directed actions performed in an atypical way, and likewise people with motor impairments can map others’ actions according to their actual motor possibilities.

## Data Availability Statement

The datasets generated for this study are available on request to the corresponding author.

## Ethics Statement

The studies involving human participants were reviewed and approved by Ethical Committee of the University of Padova. The patients/participants provided their written informed consent to participate in this study. Written informed consent was obtained from the individual(s) for the publication of any potentially identifiable images or data included in this article.

## Author Contributions

SB, MD, UC and LS designed the research. SB and MD built the experimental set up. SB and MD analyzed the data and performed the statistical analyses. All authors discussed the results, contributed to the writing, reviewed and edited the manuscript.

## Conflict of Interest

The authors declare that the research was conducted in the absence of any commercial or financial relationships that could be construed as a potential conflict of interest.

## References

[B1] Bernstein N. (1967). The Co-ordination and Regulation of Movement. Oxford, England: Pergamon.

[B2] Betti S. Castiello U. Sartori L. (2015). Kick with the finger: symbolic actions shape motor cortex excitability. Eur. J. Neurosci. 42, 2860–2866. 10.1111/ejn.13067 26354677

[B3] Brasil-Neto J. P. Cohen L. G. Panizza M. Nilsson J. Roth B. J. Hallett M. (1992). Optimal focal transcranial magnetic activation of the human motor cortex: effects of coil orientation, shape of the induced current pulse and stimulus intensity. J. Clin. Neurophysiol. 9, 132–136. 10.1097/00004691-199201000-00014 1552001

[B4] Brass M. Schmitt R. M. Spengler S. Gergely G. (2007). Investigating action understanding: inferential processes versus action simulation. Curr. Biol. 17, 2117–2121. 10.1016/j.cub.2007.11.057 18083518

[B5] Buccino G. Lui F. Canessa N. Patteri I. Lagravinese G. Benuzzi F.. (2004). Neural circuits involved in the recognition of actions performed by nonconspecifics: an fMRI study. J. Cogn. Neurosci. 16, 114–126. 10.1162/089892904322755601 15006041

[B6] Castiello U. (1997). Arm and mouth coordination during the eating action in humans: a kinematic analysis. Exp. Brain Res. 115, 552–556. 10.1007/pl00005726 9262211

[B7] Castiello U. Dadda M. (2018). A review and consideration on the kinematics of reach-to-grasp movements in macaque monkeys. J. Neurophysiol. 121, 188–204. 10.1152/jn.00598.2018 30427765

[B8] Castiello U. Stelmach G. E. (1993). Generalized representation of handwriting: evidence of effector independence. Acta Psychol. Amst. 82, 53–68. 10.1016/0001-6918(93)90004-b 8475776

[B9] Castiello U. Bennett K. M. Egan G. F. Tochon-Danguy H. J. Kritikos A. Dunai J. (1999). Human inferior parietal cortex “programs” the action class of grasping. Cogn. Syst. Res. 1, 89–97. 10.1016/s1389-0417(99)00011-x

[B10] Cattaneo L. Voss M. Brochier T. Prabhu G. Wolpert D. M. Lemon R. N. (2005). A cortico-cortical mechanism mediating object-driven grasp in humans. Proc. Natl. Acad. Sci. U S A 102, 898–903. 10.1073/pnas.0409182102 15642941 PMC545569

[B11] Cavallo A. Sartori L. Castiello U. (2011). Corticospinal excitability modulation to hand muscles during the observation of appropriate versus inappropriate actions. Cogn. Neurosci. 2, 83–90. 10.1080/17588928.2010.533163 24168477

[B12] Dempsey-Jones H. Wesselink D. B. Friedman J. Makin T. R. (2019). Organized toe maps in extreme foot users. Cell Rep. 28, 2748.e4–2756.e4. 10.1016/j.celrep.2019.08.027 31509738 PMC6899508

[B13] Finisguerra A. Maffongelli L. Bassolino M. Jacono M. Pozzo T. D’Ausilio A. (2015). Generalization of motor resonance during the observation of hand, mouth and eye movements. J. Neurophysiol. 114, 2295–2304. 10.1152/jn.00433.2015 26289463 PMC4609760

[B14] Gazzola V. Rizzolatti G. Wicker B. Keysers C. (2007a). The anthropomorphic brain: the mirror neuron system responds to human and robotic actions. Neuroimage 35, 1674–1684. 10.1016/j.neuroimage.2007.02.003 17395490

[B15] Gazzola V. van der Worp H. Mulder T. Wicker B. Rizzolatti G. Keysers C. (2007b). Aplasics born without hands mirror the goal of hand actions with their feet. Curr. Biol. 17, 1235–1240. 10.1016/j.cub.2007.06.045 17629484

[B16] Gentilucci M. Benuzzi F. Gangitano M. Grimaldi S. (2001). Grasp with hand and mouth: a kinematic study on healthy subjects. J. Neurophysiol. 86, 1685–1699. 10.1152/jn.2001.86.4.1685 11600632

[B17] Heed T. Beurze S. M. Toni I. Röder B. Medendorp W. P. (2011). Functional rather than effector-specific organization of human posterior parietal cortex. J. Neurosci. 31, 3066–3076. 10.1523/jneurosci.4370-10.2011 21414927 PMC6623762

[B18] Heed T. Leone F. T. M. Toni I. Medendorp W. P. (2016). Functional versus effector-specific organization of the human posterior parietal cortex: revisited. J. Neurophysiol. 116, 1885–1899. 10.1152/jn.00312.2014 27466132 PMC5144691

[B19] Jastorff J. Begliomini C. Fabbri-Destro M. Rizzolatti G. Orban G. A. (2010). Coding observed motor acts: different organizational principles in the parietal and premotor cortex of humans. J. Neurophysiol. 104, 128–140. 10.1152/jn.00254.2010 20445039

[B20] Keele S. W. (1981). “Behavioral analysis of movement,” in Handbook of physiology, ed. Brooks V. B. (Baltimore, MD: American Physiological Society), 1391–1414.

[B21] Lorey B. Naumann T. Pilgramm S. Petermann C. Bischoff M. Zentgraf K.. (2014). Neural simulation of actions: effector- versus action-specific motor maps within the human premotor and posterior parietal area? Hum. Brain Mapp. 35, 1212–1225. 10.1002/hbm.22246 23427116 PMC6869544

[B22] Macfarlane N. B. W. Graziano M. S. A. (2009). Diversity of grip in macaca mulatta. Exp. Brain Res. 197, 255–268. 10.1007/s00221-009-1909-z 19565227

[B23] Matelli M. Luppino G. Rizzolatti G. (1985). Patterns of cytochrome oxidase activity in the frontal agranular cortex of the macaque monkey. Behav. Brain Res. 18, 125–136. 10.1016/0166-4328(85)90068-3 3006721

[B24] Merton P. A. (1972). How we control the contraction of our muscles. Sci. Am. 226, 30–37. 10.1038/scientificamerican0572-30 4260739

[B25] Mills K. R. Boniface S. J. Schubert M. (1992). Magnetic brain stimulation with a double coil: the importance of coil orientation. Electroencephalogr. Clin. Neurophysiol. Potentials Sect. 85, 17–21. 10.1016/0168-5597(92)90096-t 1371739

[B26] Norman G. (2010). Likert scales, levels of measurement and the “laws” of statistics. Adv. Health Sci. Educ. Theory Pract. 15, 625–632. 10.1007/s10459-010-9222-y 20146096

[B27] Oldfield R. C. (1971). The assessment and analysis of handedness: the edinburgh inventory. Neuropsychologia 9, 97–113. 10.1016/0028-3932(71)90067-4 5146491

[B28] Osiurak F. Lesourd M. Delporte L. Rossetti Y. (2018). Tool use and generalized motor programs: we all are natural born poly-dexters. Sci. Rep. 8:10429. 10.1038/s41598-018-28759-2 29993002 PMC6041280

[B29] Parma V. Roverato R. Ghirardello D. Bulgheroni M. Tirindelli R. Castiello U. (2011). When flavor guides motor control: an effector independence study. Exp. Brain Res. 212, 339–346. 10.1007/s00221-011-2733-9 21618038

[B30] Peeters R. Simone L. Nelissen K. Fabbri-Destro M. Vanduffel W. Rizzolatti G.. (2009). The representation of tool use in humans and monkeys: common and uniquely human features. J. Neurosci. 29, 11523–11539. 10.1523/jneurosci.2040-09.2009 19759300 PMC6665774

[B31] Raibert M. H. (1997). Motor Control and Learning by the State Space Model. Cambridge, MA: Massachusetts Institue of Technology.

[B32] Rijntjes M. Dettmers C. Büchel C. Kiebel S. Frackowiak R. S. J. Weiller C. (1999). A blueprint for movement: functional and anatomical representations in the human motor system. J. Neurosci. 19, 8043–8048. 10.1523/jneurosci.19-18-08043.1999 10479704 PMC6782473

[B33] Rizzolatti G. Camarda R. Fogassi L. Gentilucci M. Luppino G. Matelli M. (1988). Functional organization of inferior area 6 in the macaque monkey. Exp. Brain Res. 71, 491–507. 10.1007/bf00248742 3416965

[B34] Rosenbaum D. A. (1990). Human Motor Control. New York, NY, USA: Academic Press.

[B35] Rossi S. Hallett M. Rossini P. M. Pascual-Leone A. (2009). Safety, ethical considerations and application guidelines for the use of transcranial magnetic stimulation in clinical practice and research. Clin. Neurophysiol. 120, 2008–2039. 10.1016/j.clinph.2009.08.016 19833552 PMC3260536

[B36] Rossini P. M. Barker A. T. Berardelli A. Caramia M. D. Caruso G. Cracco R. Q.. (1994). Non-invasive electrical and magnetic stimulation of the brain, spinal cord and roots: basic principles and procedures for routine clinical application. Report of an IFCN committee. Electroencephalogr. Clin. Neurophysiol. 91, 79–92. 10.1016/0013-4694(94)90029-9 7519144

[B37] Schmidt R. A. Lee T. D. Winstein C. Wulf G. Zelaznik H. N. (1988). Motor Control and Learning: A Behavioral Emphasis. Champaign, IL: Human Kinetics.

[B38] Senna I. Bolognini N. Maravita A. (2014). Grasping with the foot: goal and motor expertise in action observation. Hum. Brain Mapp. 35, 1750–1760. 10.1002/hbm.22289 23671004 PMC6869580

[B39] Sullivan G. M. Artino A. R. (2013). Analyzing and interpreting data from likert-type scales. J. Grad. Med. Educ. 5, 541–542. 10.4300/jgme-5-4-18 24454995 PMC3886444

[B40] Urgesi C. Maieron M. Avenanti A. Tidoni E. Fabbro F. Aglioti S. M. (2010). Simulating the future of actions in the human corticospinal system. Cereb. Cortex 20, 2511–2521. 10.1093/cercor/bhp292 20051359

[B41] Wright C. E. (1990). “Generalized motor programs: reexamining claims of effector independence in writing,” in Attention and Performance XIII: Motor Representation and control, ed. Jeannerod M. (Hillsdale, NJ, USA: Lawrence Erlbaum Associates, Inc.), 294–320.

